# Neurofeedback training of executive function in autism spectrum disorder: distinct effects on brain activity levels and compensatory connectivity changes

**DOI:** 10.1186/s11689-024-09531-2

**Published:** 2024-04-11

**Authors:** Daniela Jardim Pereira, Sofia Morais, Alexandre Sayal, João Pereira, Sofia Meneses, Graça Areias, Bruno Direito, António Macedo, Miguel Castelo-Branco

**Affiliations:** 1 Neurorradiology Functional Area, Imaging Department, Coimbra Hospital and University Center, Coimbra, Portugal; 2 Coimbra Institute for Biomedical Imaging and Translational Research (CIBIT), University of Coimbra, Coimbra, Portugal; 3 Faculty of Medicine, University of Coimbra, Coimbra, Portugal; 4 Institute of Nuclear Sciences Applied to Health (ICNAS), University of Coimbra, Coimbra, Portugal; 5 Psychiatry Department, Coimbra Hospital and University Center, Coimbra, Portugal; 6 Siemens Healthineers Portugal, Lisboa, Portugal; 7 Psychology Department, Coimbra Hospital and University Center, Coimbra, Portugal; 8 IATV-Instituto do Ambiente, Tecnologia e Vida (IATV), Coimbra, Portugal

**Keywords:** Neurofeedback, Dorsolateral prefrontal cortex, Autism spectrum disorders, Rt-fMRI

## Abstract

**Background:**

Deficits in executive function (EF) are consistently reported in autism spectrum disorders (ASD). Tailored cognitive training tools, such as neurofeedback, focused on executive function enhancement might have a significant impact on the daily life functioning of individuals with ASD. We report the first real-time fMRI neurofeedback (rt-fMRI NF) study targeting the left dorsolateral prefrontal cortex (DLPFC) in ASD.

**Methods:**

Thirteen individuals with autism without intellectual disability and seventeen neurotypical individuals completed a rt-fMRI working memory NF paradigm, consisting of subvocal backward recitation of self-generated numeric sequences. We performed a region-of-interest analysis of the DLPFC, whole-brain comparisons between groups and, DLPFC-based functional connectivity.

**Results:**

The ASD and control groups were able to modulate DLPFC activity in 84% and 98% of the runs. Activity in the target region was persistently lower in the ASD group, particularly in runs without neurofeedback. Moreover, the ASD group showed lower activity in premotor/motor areas during pre-neurofeedback run than controls, but not in transfer runs, where it was seemingly balanced by higher connectivity between the DLPFC and the motor cortex. Group comparison in the transfer run also showed significant differences in DLPFC-based connectivity between groups, including higher connectivity with areas integrated into the multidemand network (MDN) and the visual cortex.

**Conclusions:**

Neurofeedback seems to induce a higher between-group similarity of the whole-brain activity levels (including the target ROI) which might be promoted by changes in connectivity between the DLPFC and both high and low-level areas, including motor, visual and MDN regions.

**Supplementary Information:**

The online version contains supplementary material available at 10.1186/s11689-024-09531-2.

## Background

Autism spectrum disorders (ASD) are life-long conditions defined by multicontextual deficits in social communication and interaction and restricted/repetitive patterns of behavior, interests, or activities (DSM-5, American Psychiatric Association, 2013). Deficits in executive functions (EF) are consistently reported in ASD in several subdomains, such as concept formation, cognitive flexibility, fluency, action planning, response inhibition, and working memory (spatial and verbal) [1–4]. EF is an “umbrella term”, that covers all these aforementioned multiple high-order cognitive processes and sub-processes, being responsible for guiding and managing cognitive, emotional, and behavioral functions, in a goal-direct fashion. It mediates learning and regulation of stress and emotions, with high individual, social, and economic impact [5]. Specifically, the working memory subdomain represents the ability to store (short-term memory), process, and manipulate information. Based on several studies reporting lower performance on EF tests in individuals with ASD, particularly set-shifting, response inhibition, and working memory, Russell and colleagues [6] proposed the executive dysfunction theory. Although it is debatable if this represents a core feature in ASD, and despite the heterogeneity of EF dysfunction, this model seems to have a broad impact on the ASD phenotype [7]. EF dysfunction accounts for both core social aspects and non-core complex motor features in ASD [3] and deeply influences developmental outcomes and daily life functioning [1, 7, 8].

For several years, the anatomic and functional correlations, mainly based on brain lesions, pointed out the prefrontal cortex as responsible for executive functions [9]. The dorsolateral prefrontal cortex (DLPFC), in particular, is involved in several executive domains such as abstract reasoning, response inhibition, planning, cognitive flexibility, and working memory [10]. Specifically in working memory, DLPFC monitors and actively manipulates the information, performing the necessary updates [11]. DLPFC is a functionally defined area, parcellated at cytoarchitectonic and anatomic levels, comprising at least two Broadmann areas [8, 12], mainly centered on the middle frontal gyrus (MFG). Recent research suggests that DLPFC also has functional subregions connected with different brain areas, based on functional and structural connectivity data [13]. Thus, it is now widely accepted that the executive functions not solely depend on the prefrontal cortex, but on its interaction with various cortical and subcortical structures, namely the superior parietal cortex, pre-supplementary motor area (SMA), anterior cingulate cortex (ACC), thalamus, and basal ganglia [11]. These regions integrate the superordinate fronto-cingulo-parietal network that underlies EF processing, in which the DLPFC acts as a key hub [9, 11, 14]. More recently, an extended multi-demand network (MDN) was shown to be consistently recruited in working memory, attention, and inhibition, including as core regions the MFG, pre-SMA, and anterior insula [15].

In ASD, functional magnetic resonance imaging (fMRI) studies targeting EF showed inconsistent results, which were addressed in a recent meta-analysis considering three EF processes: inhibition, switching, and updating [16]. They found a common brain activation pattern in ASD across EF processes, including the bilateral inferior frontal gyri (IFG), left precentral gyrus, right superior medial frontal gyrus, and left inferior parietal gyrus [16]. Furthermore, when compared to controls, they found higher spatial overlapping, across independent fMRI studies, in the left inferior parietal gyrus. Lower overlap was found in the right IFG, right ACC, left SMA, and right middle frontal gyrus (MFG).

Zhang Z. et al [16] Wide disruption in functional connectivity, also affecting dorsal attention and executive-related networks, has been reported in ASD. A large database study showed overconnectivity in unimodal sensory networks (except medial visual in the occipital lobe) and predominant underconnectivity in supramodal high-level networks (except default mode network and dorsal attention) [17]. Specifically, in working memory, a more posterior and right-lateralized pattern of activation and functional connectivity was reported in ASD [18].

More recently, neuroimaging has also emerged as a potential therapeutic tool in neuropsychiatric conditions with the implementation of real-time fMRI (rt-fMRI) for neurofeedback. The rationale of neurofeedback is that self-modulation of brain activity, targeting a specific area or network, will promote learning through association, allow the internal perception of cognitive strategies that result in improvement and, ultimately, induce functional and/or structural persistent neural changes (neuroplasticity) [19]. To our knowledge, three rt-fMRI neurofeedback studies in autism have been published thus far, all targeting areas involved in social cognition, particularly face processing. In the first study, Ramot et al., (2017) targeted the covert (nonvolitional neurofeedback) training of aberrant networks in ASD, with the feedback signal based on connectivity metrics between the superior temporal sulcus (STS) and the somatosensory cortex. Neurofeedback training-induced long-term functional changes in the target regions as well as at the network level, correlated with changes in behavior in the clinical group [20]. Second, Direito et al., (2021) similarly targeted STS through an interface based on the dynamic morphing expression of an avatar using mental imagery of facial expressions as a task. The authors reported improvements with neurofeedback in neuropsychological measures (emotion recognition, adaptive behavior, and mood) persisting for 6 months [21]. The third study also demonstrated the ability for self-modulation with neurofeedback in five individuals with ASD, this time targeting the fusiform face area (FFA), which is closely related to the STS for face processing, although not a part of the social brain [22].

Although social cognition has emerged so far as an attractive target for neuromodulation in ASD, we consider that approaching executive dysfunction [6] might have an equally relevant impact on the challenges ASD individuals face across their lifespan [1, 23, 24].

Taking into account the aforementioned anatomo-functional knowledge of the executive network, we conclude that the DLPFC is a suitable candidate for the target ROI. The DLPFC is a key hub in EF, and its modulation has the potential to result in a significant functional impact in the network [25], as previously reported in neurotypicals [25–28], without the technical difficulties posed by connectivity-based neurofeedback [29, 30].

In this study, we apply a neurofeedback protocol for executive functions training, for the first time in ASD, targeting the left DLPFC while performing a verbal working memory imagery task. First, we aim to prove that individuals with autism are able to modulate the DLPFC through neurofeedback. Second, we hypothesize that neurofeedback mediates an increased ability of patients to modulate the target region activity associated with the reorganization of the network seeded on the DLPFC.

## Methods

### Participants

A total of 13 male participants (age: M=22.83 years, SD= 4.5, range 18-32), diagnosed with autism, without intellectual disability (Full-Scale Intelligence Quotient (FSIQ) superior to 80; FSIQ: M = 109, SD= 10.7 [31]), were recruited to this study. ASD diagnosis was established based on: 1) clinical evaluation by a psychiatrist (SM) with expertise in neurodevelopmental disorders, considering the current diagnostic criteria for ASD from the Diagnostic and Statistical Manual of Mental Disorders 5^th^ edition [32], and 2) an ASD cutoff score from social and communication symptoms on Module 4 of the Autism Diagnostic Observation Schedule (ADOS-2) (M=14, SD=5.4), administered by an experienced psychologist (GA) [33]. Three patients were on stable antidepressant and antipsychotic medication, which they followed as usual on the day of the experiment. One participant was excluded from the data analysis due to significant structural abnormalities diagnosed during MR acquisition (compensated congenital obstructive hydrocephalus, a consequence of an aqueductal web).

Additionally, 17 neurotypical volunteers (10 male, age: M=27.8 years, SD=4.2, range 22-38) composed the control group. All were graduate students or had graduate/postgraduate degrees. They had no history of neurological or psychiatric conditions.

All participants had normal or corrected-to-normal vision and were right-handed. All gave informed written consent before participating, in accordance with the Declaration of Helsinki, and the study complied with the safety guidelines for magnetic resonance imaging (MRI) research on humans. The work was approved by the Ethics Committee of the Faculty of Medicine of the University of Coimbra and Coimbra Hospital and University Centre. For additional details on clinical and demographic information see Supplementary Material.

### MR data acquisition

Data acquisition was initially conducted on a 3T Siemens Magnetom TrioTim scanner with a 12-channel head coil, which was upgraded during the period of this study for a Magnetom Prisma^fit^ scanner with a 64-channel head coil. The scanning session started with an anatomical high-resolution magnetization-prepared rapid acquisition gradient echo (MPRAGE) sequence (176/192 slices; echo time (TE): 3.42/3.5 ms; repetition time (TR): 2530 ms; voxel size: 1mm^3^ isotropic; flip angle (FA): 7°; field of view (FOV): 256 × 256 mm) for coregistration with functional data. Functional imaging was acquired with an echo-planar imaging (EPI) sequence (32 slices, in-plane resolution: 3 × 3 mm, FOV: 192 × 210 mm, matrix 64 x 70, slice thickness: 2.5 mm, FA: 75°, TR = 2000 ms and TE = 30 ms). We also performed additional structural images at the end of the neurofeedback session, at least including a T2 FLAIR and a T2 TSE, which were evaluated by an expert neuroradiologist to exclude significant structural brain abnormalities.

### Experimental protocol

The rt-fMRI neurofeedback protocol was previously validated with a sham-controlled study in a neurotypical population [34], where we reported the experimental procedure in detail (please refer to the Methods section of the previous paper). However, for readability, the protocol is briefly described here. The CRED-nf checklist [35] is provided as Additional file 1.

**Fig. 1:**
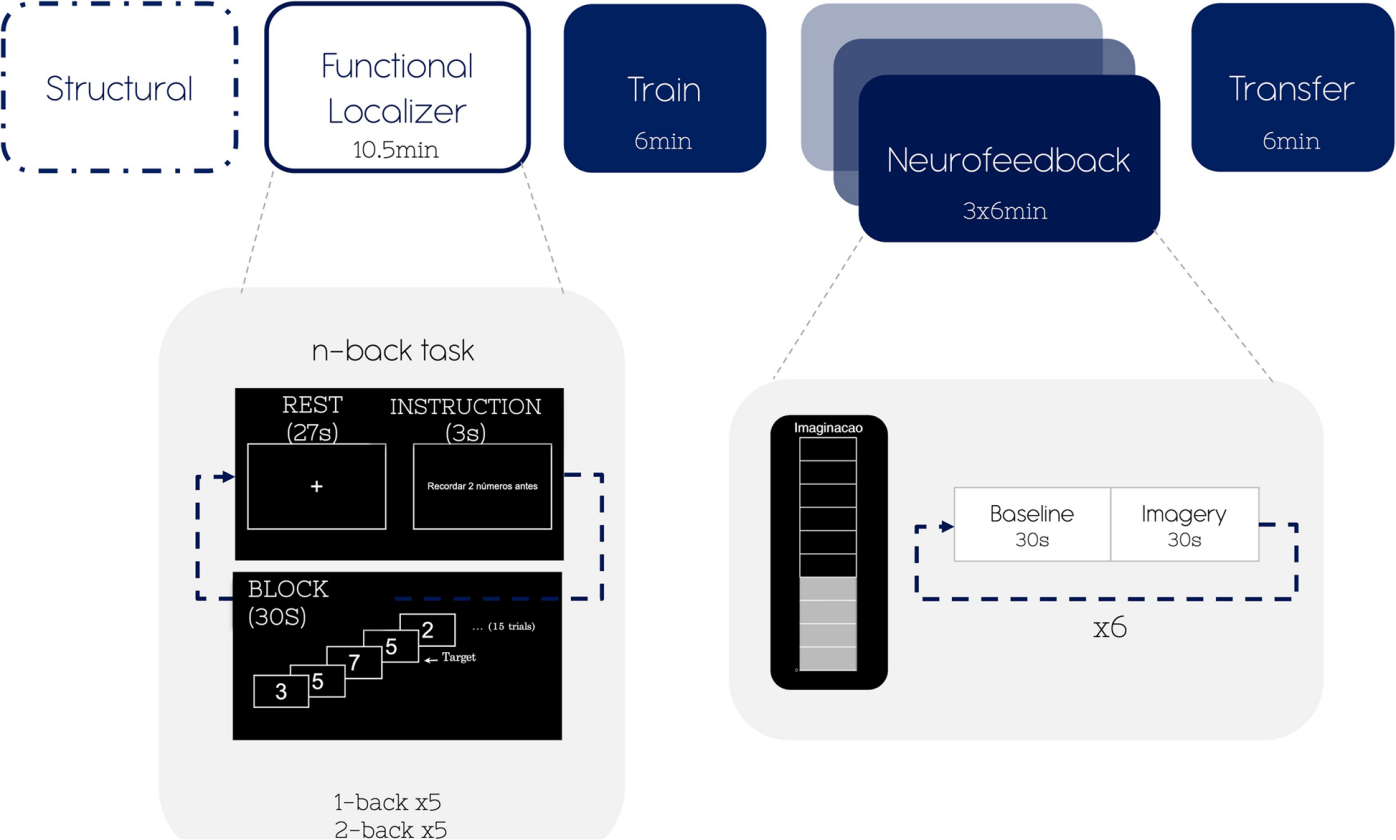
Schematic representation of the neurofeedback protocol

The experimental design consists of six functional runs, as represented in Fig. 1. First, a localizer run was acquired to functionally map the left DLPFC, followed by five imagery runs. The first (pre-neurofeedback) and the last (transfer) imagery runs were performed without providing feedback to the participant (empty thermometer), although the participant was instructed to perform the exact same imagery task. The scanning session lasted approximately 1.5 hours, followed by a debriefing questionnaire.

### Functional localizer

The functional localizer consisted of an n-back task, a well-known test for verbal working memory, including two task conditions (‘1-back’ and ‘2-back’) randomly distributed in 10 blocks (5 blocks each) alternating with ‘Baseline’ blocks (total length: 10.5 minutes). During each task block, the screen displayed sequences of 15 digits, and the participant was instructed to press a button when the number displayed matched the one presented immediately before in the 1-back condition or when it matched the number two steps earlier in the sequence in the 2-back condition. Each digit was displayed for 400 ms, and each sequence had 5 “target” digits. This stimulus was created and presented in Presentation 20.1 (Neurobehavioral Systems, Inc).

Based on these data, we functionally individually defined the DLPFC online using the real-time fMRI software package Turbo-BrainVoyager 3.2 (TBV; Brain Innovation, Maastricht, The Netherlands). We generally considered ROIs appropriate for NF targets when PSC was at least 1%. Anatomical references were also taken into account by an expert neuroradiologist (DJP) to determine the DLPFC, guaranteeing that it was located anterior to the premotor cortex and above the planes of the lateral ventricles (roughly including the middle frontal gyrus – Brodmann area 46). All targets were selected on the left hemisphere since participants were performing a verbal working memory task during imagination runs and we expect left lateralization of the DLPFC, as observed in language-related hemisphere dominance in right-handed subjects [36]. A probability map for the target ROIs selected online for all subjects is represented in Fig, 2.

**Fig. 2:**
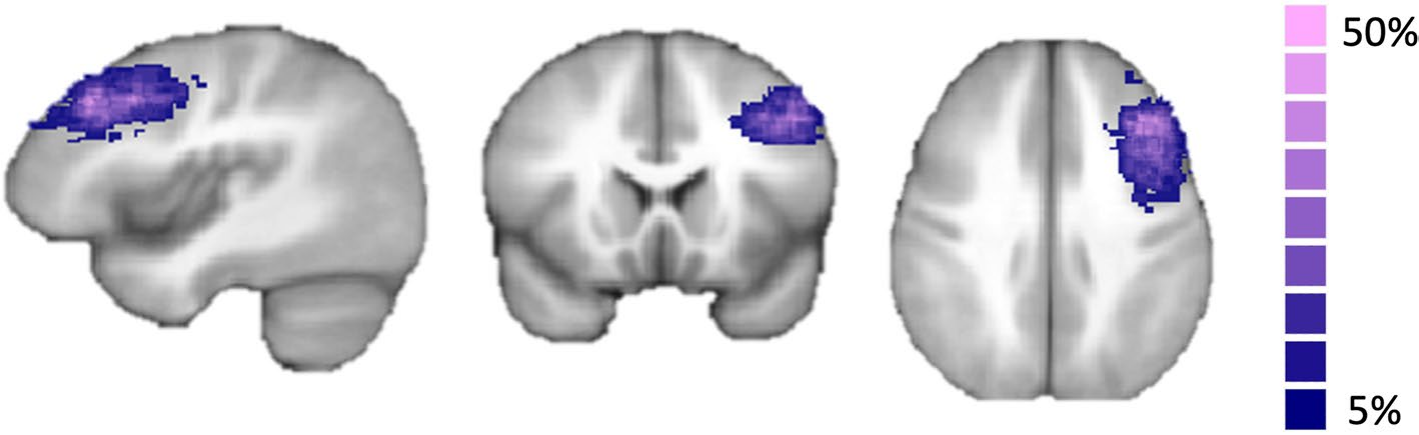
Probability map of the target ROI selected online in the DLPFC for all participants (maximum probability = 44.82%)

### Imagery runs

The imagery runs included two conditions - ‘Imagery’ and ‘Baseline’ - presented alternatively 6 times per run with an additional ‘Baseline’ block at the beginning of each run, with each condition block lasting 30 seconds.

Visual feedback was provided in the form of a thermometer that was updated every TR based on the mean ROI activation of the neurofeedback target selected during the localizer run for each participant. The thermometer was divided into 10 discrete levels with a maximum value of 2.5%, where each level represented a given range of percent BOLD signal change (0 for an empty thermometer and 0.25% for each level). The feedback value fb for the current time point n is calculated within each block given the current value val, a baseline level bl (mean BOLD value in the target region, during previous ‘baseline’ block) according to Equation:

1 $$\boldsymbol{fb}\ \left(\textrm{n}\right)=\frac{\textrm{val}\left(\textrm{n}\right)-\textrm{bl}}{\textrm{bl}}\ \times\ 100$$

During the neurofeedback runs, participants were instructed to empty the thermometer during ‘Baseline’ conditions and increase the thermometer bars during the ‘Imagery’ condition. As a cognitive strategy to increase the number of bars in the thermometer, we recommended that the participants repeatedly try to generate a sequence and then recite it backward subvocally [37], adjusting the content, length and difficulty of the sequences according to the feedback.

### Debriefing

After the scanning session, participants answered a debriefing questionnaire that included subjective questions about their feelings during the acquisition (*How did you feel during the neurofeedback session?*), the contingency between effort and feedback change (*Did you feel there was a correspondence between the strategies used and the given feedback?*) and the cognitive strategies used (*What was the maximum number of sequences you could picture in each block? And the maximum digit number? Which strategies worked better? And which ones did not work?*).

### fMRI data analysis for target ROI GLM and whole-brain comparisons

Offline fMRI data analysis was performed using BrainVoyager 22.2 (Brain Innovation, Maastricht, The Netherlands). Preprocessing steps included slice scan time correction, 3D motion correction (6 degrees of freedom), temporal high-pass filtering (GLM Fourier method, 2 cycles), spatial smoothing using a 3D Gaussian kernel (FWHM = 6 mm), and normalization to Talairach coordinate space with co-registration with the anatomical scan.

First-level analysis was performed using a standard GLM for each run. The design matrix included a predictor for each experimental condition and confound predictors for the six motion parameters (three translational and three rotational) and motion spikes (relative root mean square displacement threshold of 0.25 mm).

One of our main goals was to prove modulation of the neurofeedback target region in the ASD group (statistically significant activation) and explore the evolution along the runs both within and between groups. To this end, we performed an ROI-GLM to retrieve the ROI activation level (PSC relative to baseline) for each run, followed by a mixed ANOVA considering imagery runs (pre-neurofeedback, transfer, and each neurofeedback run) as within-subjects variables and group as a between-subject factor. To distinguish between target activity associated only with the verbal working memory imagery task and the neural effect promoted by the provided feedback, we performed independent sample t-tests separately considering the runs with and without neurofeedback. Spearman's rank correlation was computed to assess the relationship between pre-neurofeedback/basal DLPFC activity (pre-neurofeedback run) and ADOS-2 total score.

To explore whole-brain neurofeedback training-induced activation patterns, we performed a second-level analysis using a random effects (RFX) GLM and contrasting ‘Imagery’ and ‘Baseline’ conditions (FDR-corrected q=0.005). Contrasts between groups were obtained with conjunction analysis of RFX.

### Functional connectivity data analysis

Data were preprocessed and analyzed using the CONN toolbox version 21a [38]. The preprocessing pipeline comprised functional realignment, slice-timing correction, outlier identification, direct normalization into MNI space, segmentation into gray matter, white matter, and CSF and spatial smoothing with a Gaussian kernel of 6 mm full-width half maximum (FWHM). Physiological artifacts and residual subject movement effects were removed through a combination of linear regression of potential confounding effects in the BOLD signal (noise components from cerebral white matter and cerebrospinal areas, estimated subject-motion parameters, identified outlier scans and task effects) and temporal bandpass filtering (0.008Hz-0.09Hz). Due to a registration error in CONN that could not be avoided, the anatomical and functional data of two participants in the ASD group were preprocessed in fMRIPrep version 20.2.7. This process included steps equivalent to those in CONN preprocessing (motion correction, coregistration, and normalization). A complete description of the pipeline can be found in the Additional file 2. The preprocessed data were then imported into CONN and spatially smoothed.

In our first-level analysis, we implemented a weighted seed-based connectivity (wSBC) analysis, computed using a weighted least squares (WLS) linear model, with temporal weights corresponding to each task condition. The seed was defined as the subject-specific target ROI (DLPFC), normalized to MNI space, and masked for gray matter.

The second-level analysis was based on a GLM framework [for a detailed methodological explanation, see [39]]. We contrasted conditions/runs within each group (t-test) to evaluate the potential network short-term reorganization induced by neurofeedback and between-group contrasts (ASD group>control group) to explore how this process differs in ASD. Between-group contrasts were normalized in relation to the baseline to minimize differences induced by divergent MR acquisition parameters. Cluster-level inferences were based on Gaussian random field theory parametric statistics [40], using a stringent initial cluster-forming height threshold set at *p*=0.001 and an FDR-corrected *p*<0.05 cluster-level threshold to select those clusters that were significant. For additional details on experimental design and analysis see Supplementary Files (including the CRED-nf checklist).

## Results

### Modulation of DLPFC (ROI GLM analysis)

Considering the definition of successful modulation as a significant positive t-value for the contrast of interest (‘Imagery’>’ Baseline’), we found that both groups were highly proficient in modulating the target region (DLPFC) using the instructed strategy. Taking into account all imagery (pre-neurofeedback, transfer, and neurofeedback runs; 60 in the ASD group and 85 in the control group), we found statistically significant modulation of the target ROI in 50 runs (84%) in the ASD group and 83 runs (98%) in the control group. In the control group, the only two runs (from all 85 runs) without significant modulation were without feedback – one in transfer and the other in pre-neurofeedback in different individuals – meaning this group achieved modulation of DLPFC in 100% of the neurofeedback runs. In the ASD group, successful modulation was equally distributed in all runs (84% in each).

As anticipated for a single-session neurofeedback protocol, we did not find improvement in DLPFC activity from pre-neurofeedback to transfer run that demonstrates a learning effect. In contrast, DLPFC activity slightly decreased in both ASD and control samples during the transfer run, possibly due to fatigue. Additionally, considering a linear trend of DLPFC activation along NF runs, the results were mixed, with a positive trend in 6/12 individuals with ASD and 9/17 neurotypical individuals.

**Fig. 3:**
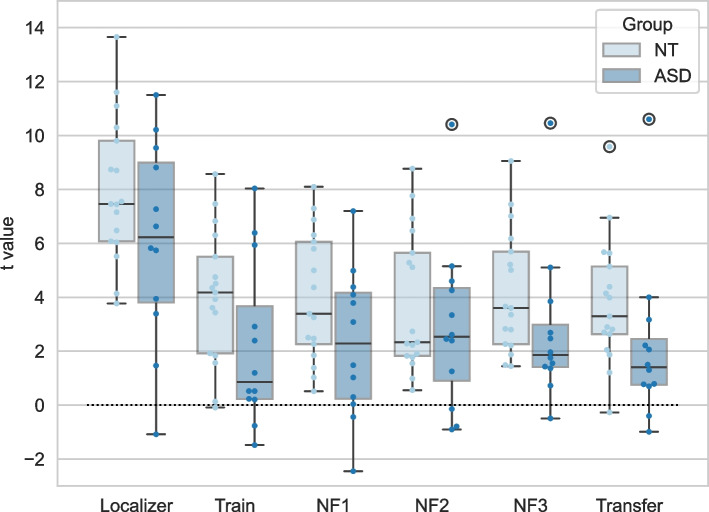
Mean T value across subjects in the target ROI (DLPFC) in neurofeedback runs for ASD and neurotypical individuals (NT). Points are laid over a 1.96 standard error of the mean (SEM) (95% confidence interval) in lighter color and a 1 standard deviation (SD) in darker color

The group mean t-value for the DLPFC in each run is represented in Fig. 3, being lower in the ASD group. The mixed ANOVA considering each imagery run showed a significant effect of the group on DLPFC activity (p=0.045). When separately considering differences between groups in runs with and without feedback, we found that activity in the target ROI was significantly lower in the ASD group (M=2.16, SD=2.21) than in the control group (M=3.92, SD=1.82), specifically when feedback was not provided (t(27)=2.353, p=0.026).

**Fig. 4:**
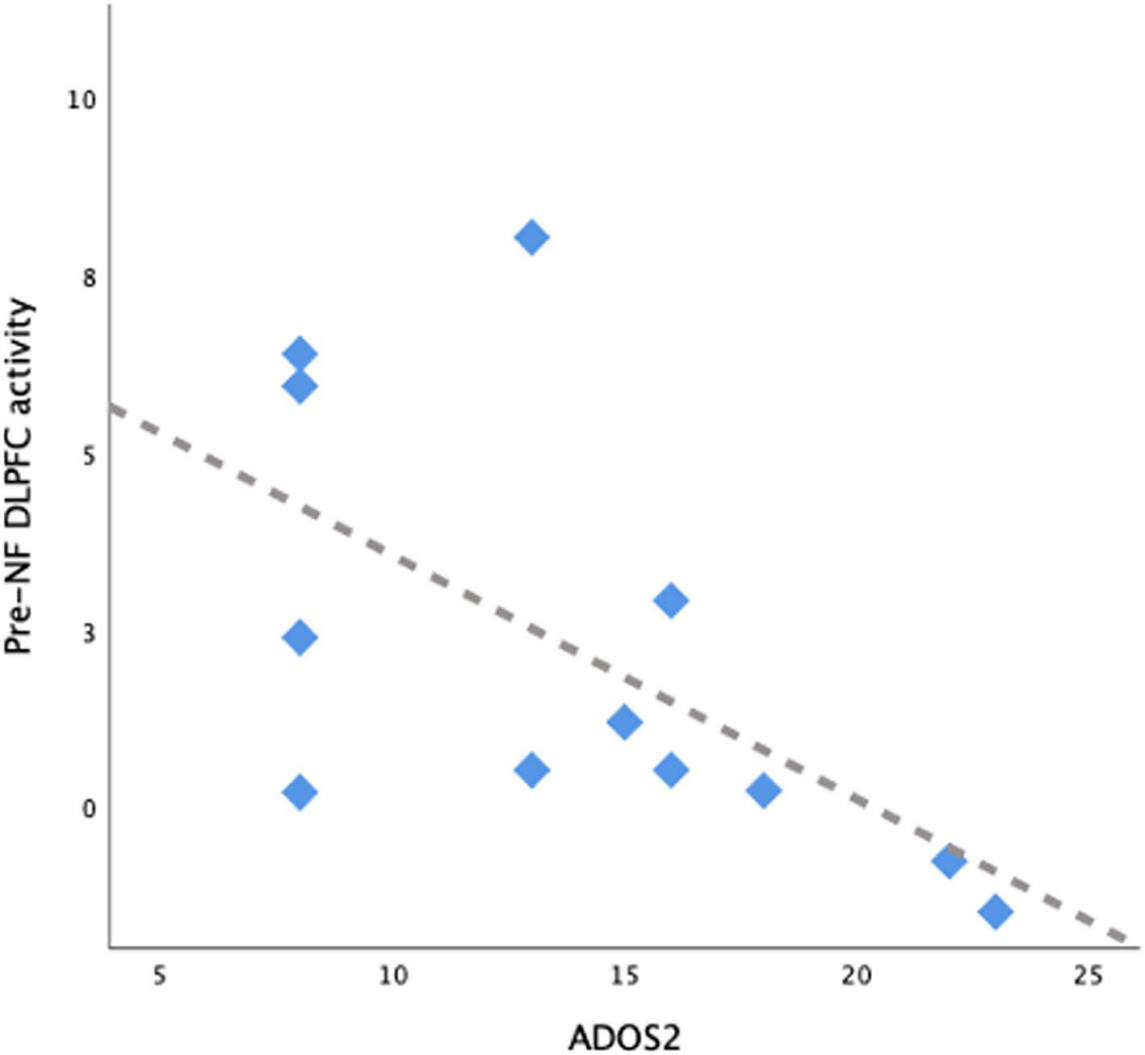
Scatterplot showing the relation between ADOS-2 (total score) and DLPFC in ASD group

We found a negative correlation between pre-neurofeedback DLPFC activity during the imagery task and ADOS-2 total score in ASD group, r(df) = -0.593 p =0.042 (Fig. 4).

### Whole-brain RFX-GLM analysis

**Fig. 5:**
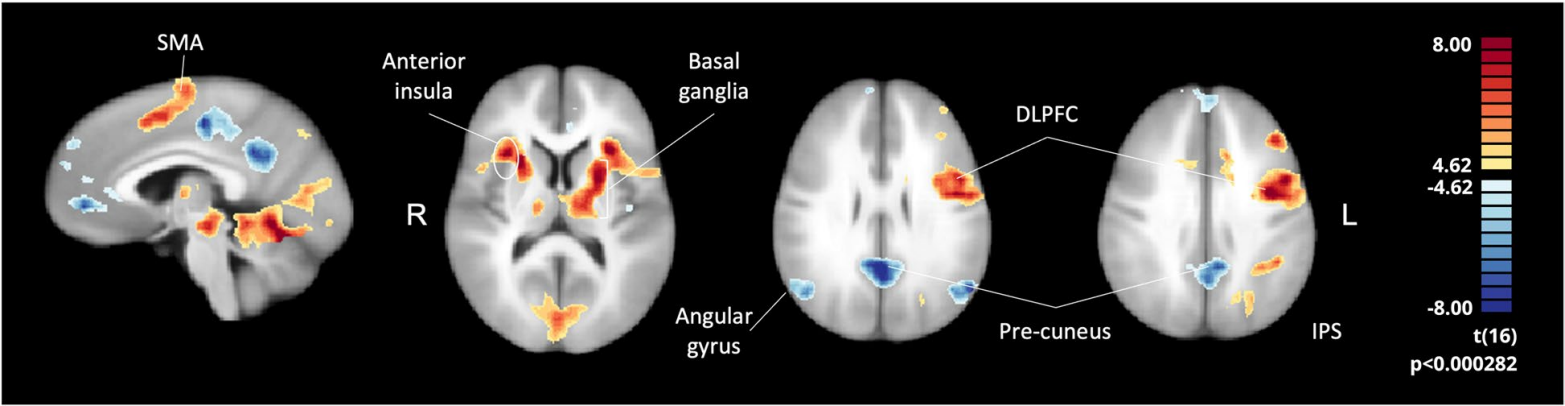
Control group RFX GLM map for all neurofeedback runs during ‘Imagery’ condition. Sagittal (x=-1, talairach coordinate) and axial (z=17 for left image and z=42 for right image, talairach coordinates) views, showing the typical pattern of activation during executive function tasks, including DLPFC, intraparietal sulcus (IPS), supplementary motor area (SMA), anterior insula, premotor cortex, inferior frontal gyrus (IFG) and basal ganglia, with deactivation of DMN (precuneus, medial frontal area and lateral parietal/angular gyrus)

The whole-brain activation map for the control group considering first the neurofeedback run is represented in Fig. 5, showing the expected pattern of activation during executive tasks, including frontoparietal areas, basal ganglia, anterior insula, premotor and supplementary motor areas, and dopaminergic structures in the brainstem. The functional map also enhances the deactivation of the default mode network (DMN), typically anti-correlated with the executive network.

**Fig. 6:**
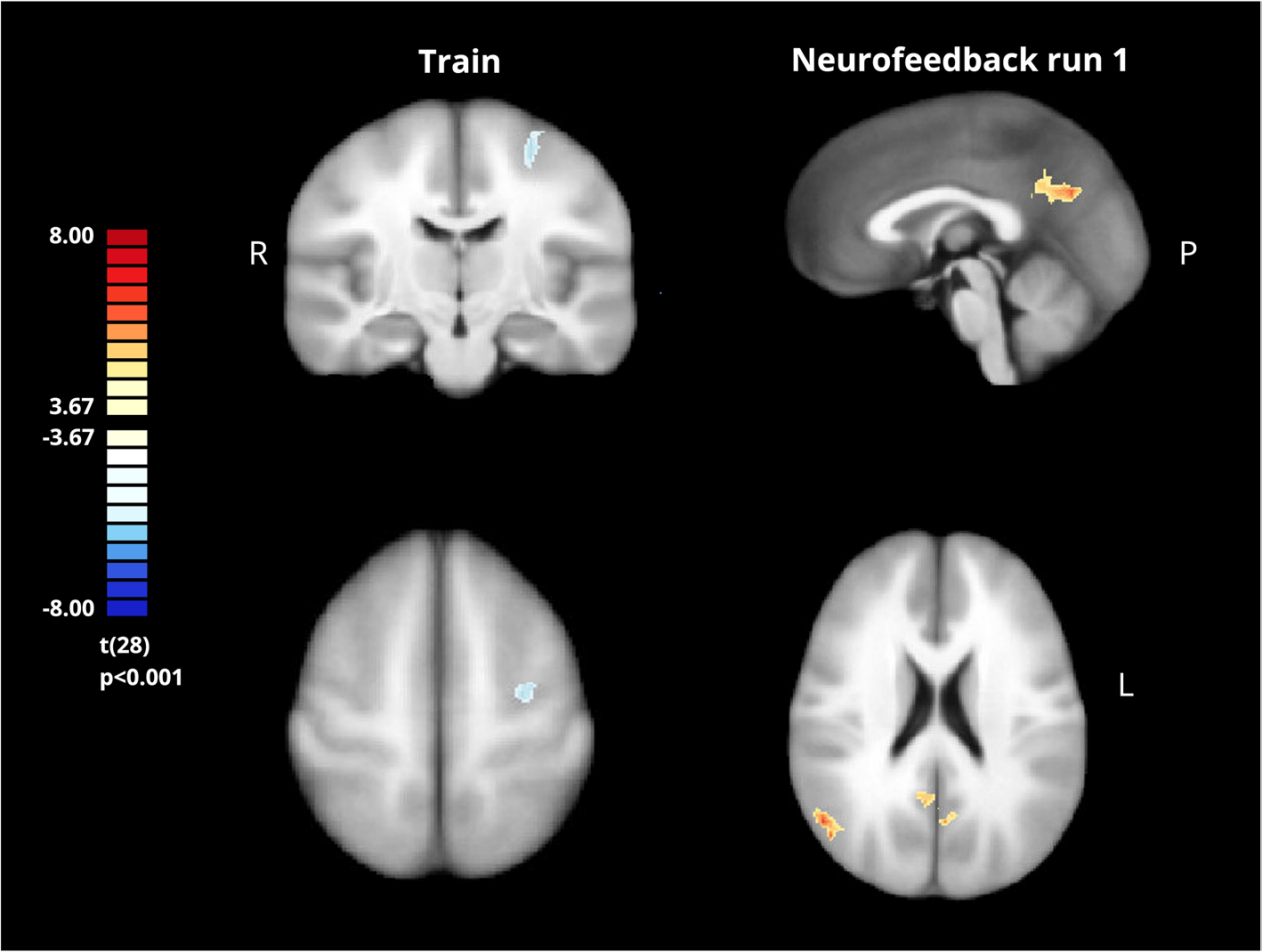
Whole-brain RFX GLM map comparing groups (ASD>NT) in pre-neurofeedback (left) and first neurofeedback conditions (right) during the ‘imagery’ condition. Coronal (left top, y=13, talairach coordinate) and axial (left bottom, z=55, talairach coordinate) views

We compared ASD and control group whole-brain activation maps, considering the contrast ‘Imagery’>’ Baseline’ during each neurofeedback run (Table 1). In the pre-neurofeedback run, we found a single cluster of lower activation in the ASD group in the premotor cortex (Fig. 6). In the first neurofeedback run, significant positive clusters emerged in the bilateral precuneus and the right lateral parietal/angular gyrus (Fig. 6). In the remaining runs with neurofeedback and the transfer run, no significant differences were observed between the ASD and control groups, suggesting that the brain activation patterns of both groups became more similar over the session.

**Table 1:** Whole-brain RFX GLM group comparison (ASD>NT) activity clusters

Group Comparison (ASD>NT)				
Run	Cluster	Peak voxel coordinates (x, y, z)	*p* value	N° of voxels
*Pre-neurofeedback*	Left premotor cortex	-27, -13, 52	0.000027	211
*Neurofeedback 1*	Right angular gyrus	51, -61, 22	0.000001	309
	Bilateral precuneus	-1, -64, 28	0.000002	1712
*Neurofeedback 2*	No significant clusters			
*Neurofeedback 3*	No significant clusters			
*Transfer*	No significant clusters

## Seed-based connectivity analysis

### Within-Group analysis

To investigate connectivity changes seeded on DLPFC related to neurofeedback in each group, we compared the ‘Imagery’ condition in 1) pre-neurofeedback *versus* the first neurofeedback run to evaluate immediate connectivity changes related to the introduction of a feedback signal in the task and, particularly, considering the results in whole brain RFX GLM analysis; and 2) transfer *versus* pre-neurofeedback to probe networks underlying learning induced by neurofeedback. Data are summarized in Table 2.

**Table 2:** Seed-Connectivity Analysis

	Contrast	Cluster	Peak voxel coordinates (x, y, z)	Cluster size FDR-corrected *p*-value	N° of voxels
*Within Group*					
* ASD*	*Transfer>Pre-neurofeedback*	Right precentral and postcentral gyri	+36, -22, 58	0.046	39
* Controls*	*Transfer >Pre-neurofeedback*	Left parahippocampal gyrus	-16, -04, -28	0.017	41
		Right parahippocampal gyrus	+10, -10, -26	0.033	31
	*First NF>Pre-neurofeedback*	Left superior frontal gyrus	-10 +56 +34	0.00011	89
		Left postcentral gyrus	-24 -44 +64	0.021	37
		Right SMA	+06 -10 +64	0.044	27
		Left medial prefrontal cortex	-02 +58 +10	0.044	27
* Between Group* * (ASD>NT)*	*Transfer*	Left middle frontal gyrus	-30, + 22, +52	0.00018	110
		Left middle temporal gyrus	-64, -58, -04	0.00022	98
		Left medial prefrontal cortex	-04, +64, +06	0.00059	81
		Right occipital pole	+24, -96, 00	0.004	54

**Fig. 7:**
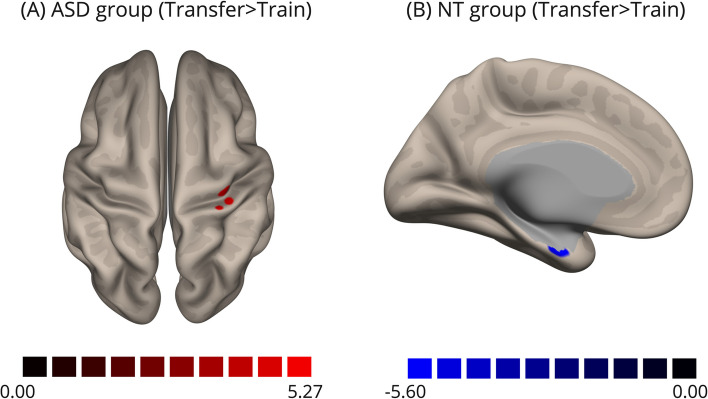
Seed-based (DLPFC) connectivity analysis, within-group comparisons transfer> pre-neurofeedback for ASD (**A**) and control groups (**B**). A–Cluster of hyperconnectivity with DLPFC in the transfer run compared to the pre-neurofeedback run located in the right pre and postcentral gyri. B-C luster of hypoconnectivity with the DLPFC in the transfer run compared to the pre-neurofeedback run located bilaterally in the parahippocampal gyri (here represented by the medial view of the left hemisphere) in the control group

In the ASD group, we found increased connectivity between the left DLPFC and right precentral and postcentral gyri during the transfer run compared to the train run (Fig. 7A). No significant differences were found in pre-neurofeedback vs. first neurofeedback runs.

**Fig. 8:**
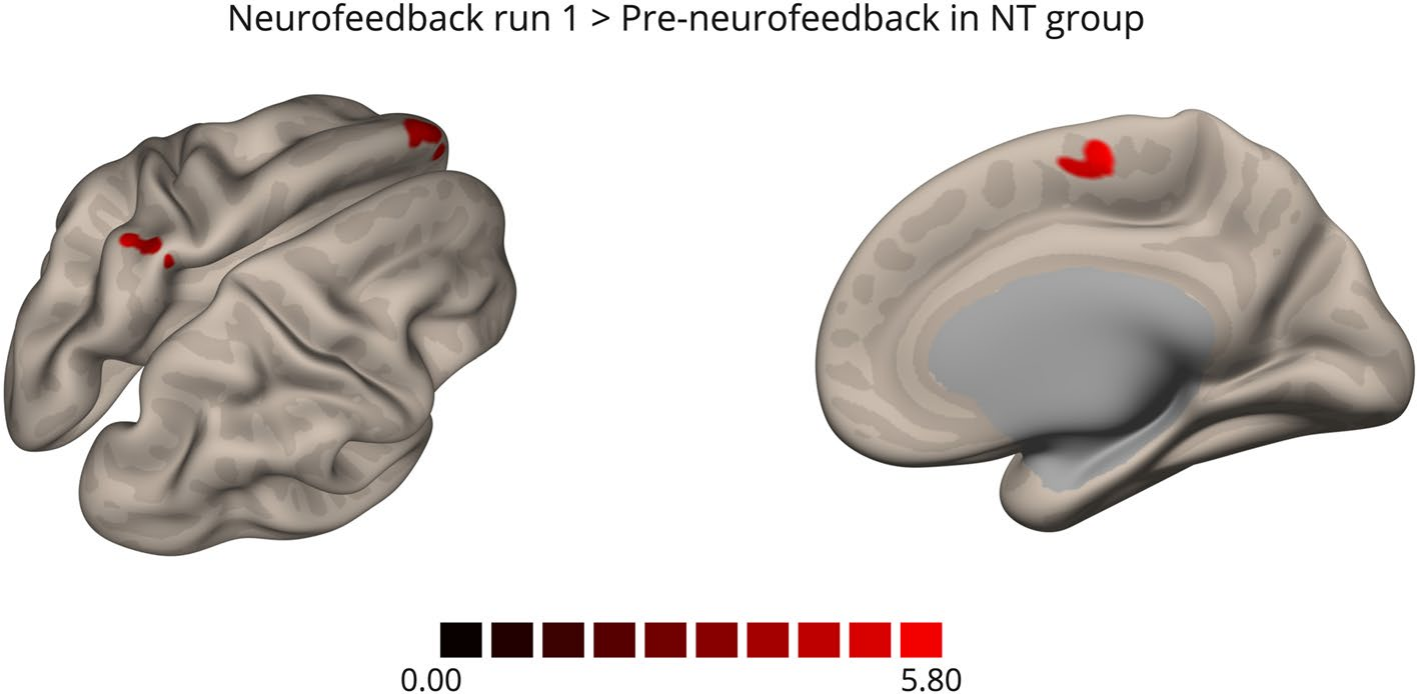
Seed-based (DLPFC) connectivity analysis, within-group comparisons first neurofeedback> pre-neurofeedback in control group. Clusters of hyperconnectivity with the DLPFC in the first neurofeedback run comparing to pre-neurofeedback run located in the left superior frontal gyrus, left ventral mPFC and left postcentral gyrus in the right superior view (left) and in the right SMA in the right medial view (right)

In the control group, the comparison between the first neurofeedback run and the pre-neurofeedback run showed increased connectivity between the left DLPFC and left superior frontal gyrus (SFG), left postcentral gyrus, right SMA, and left ventral medial prefrontal cortex (mPFC) (Fig. 8). The comparison between the transfer run and pre-neurofeedback run showed bilateral clusters at the parahippocampal gyrus (Fig. 7B).

### Between-Group analysis

**Fig. 9:**
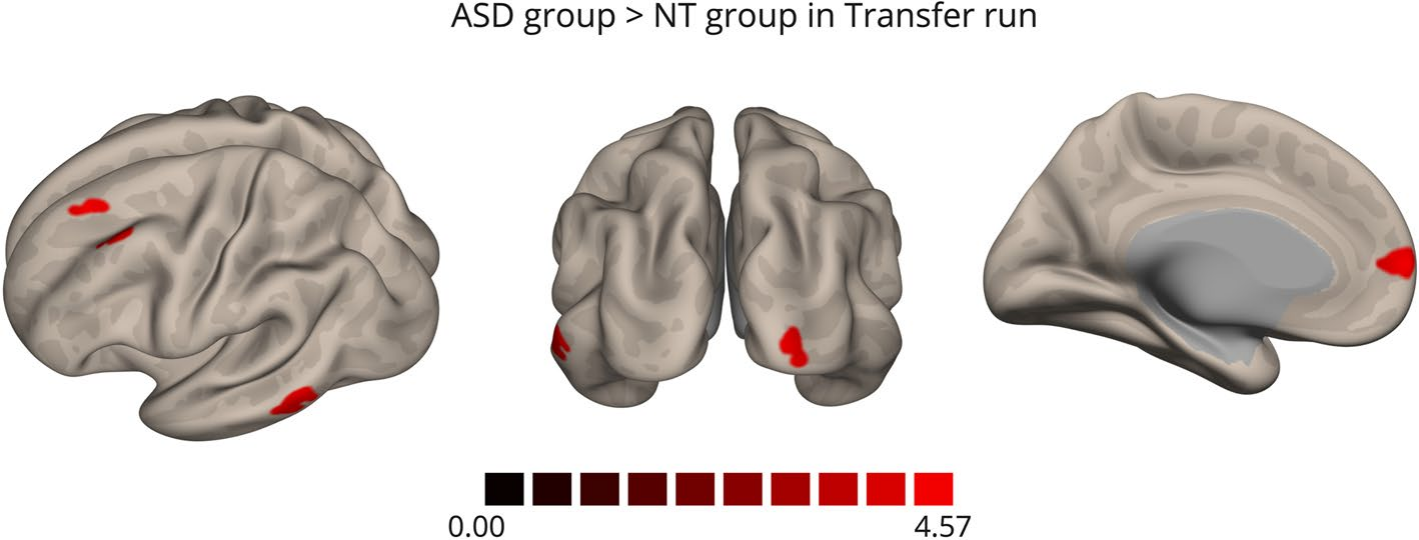
Seed-based (DLPFC) connectivity analysis between group comparison (ASD>NT) during the transfer run (‘Imagery’>’Baseline’). Clusters of hyperconnectivity with the DLPFC in the left middle frontal gyrus, left middle temporal gyrus, left ventral mPFC and right occipital pole

We only found significant functional connectivity differences between groups in the transfer run, contrasting with the absent amplitude differences reported above, with higher connectivity in the ASD group between the DLPFC and left superior and posterior middle frontal gyrus (MFG), left middle temporal gyrus (MTG), left ventral mPFC and right occipital pole (Fig. 9).

### Debriefing questionnaire

According to the debriefing questionnaire, almost all participants perceived a correspondence between the given feedback and the imagery task. Most participants followed the suggested imagery task for activating the DLPFC (inverted recall of self-generated numeric sequences) but used different strategies to increase the thermometer. For example, some participants based their strategy on mental calculation, while others relied on “visualization” of the generated sequence. In both groups, some participants reported that easier sequences (such as phone numbers, birthdays, or repeated numbers) were the more effective strategies; in contrast, other participants reported that the better way to increase the thermometer bars was increasing the task difficulty (for example, aleatory numbers, bigger sequences, thinking fast). The reported maximum number of digits and sequences per block was not significantly different between groups, with ASD individuals reporting 4 to 20 digits (M=9.375, SD=5.07) and 2 to 30 sequences (M=8.6, SD=8.9) and controls reporting 4 to 20 digits (M=8.1, SD=4.1) and 2 to 12 sequences (M=6.1, SD=3). An additional table summarizes the responses to the final debriefing questionnaire (see Additional file 3).

## Discussion

In this study, we aimed to explore working memory training through rt-fMRI neurofeedback as a potential rehabilitation tool in ASD, investigating the immediate neural effects of a single session in the target ROI, whole-brain activity patterns, and target-based functional connectivity.

First, we demonstrated the feasibility of our working memory neurofeedback paradigm, as both groups of participants were able to modulate the DLPFC with imagery, with or without feedback. Importantly, and in line with previous metanalysis in autism [41], the mean values of DLPFC percent signal change were consistently lower in all runs in our ASD sample. Importantly, differences between groups were specifically significant in runs without feedback provided (that is, pre-neurofeedback and transfer), translating an increased DLPFC activity during neurofeedback runs, with an approximation to the controls values, not elicited by imagery alone. Previous studies that applied rt-fMRI neurofeedback in clinical populations have demonstrated an increase in the activation of the target region during neurofeedback training as a proof of concept of its therapeutic effect, such as in attention-deficit/hyperactivity disorder [42], stroke [43], Huntington’s disease [44], tinnitus [45] and pain [12]. In neurofeedback experiments targeting DLPFC in neurotypicals, increased activation of DLPFC along the sessions was also understood as a positive result [37, 46]. Indeed, the majority of the studies in cognitive neuroscience, specifically in the neurofeedback literature, assume higher activation as training success [47, 48], even though there are claims in working memory research that the decrease in brain activity may suggest higher efficiency [49]. In our sample, we found a significant negative correlation between ADOS-2 total score and DLPFC activity in training, suggesting that lower target activity pre-neurofeedback was associated with greater autistic core phenotypical traits.

We did not expect the changes to be limited to the anatomical boundaries of the target ROI, as other regions are being recruited, both related to the imagery paradigm and the neurofeedback itself [19, 47, 50, 51]. Thus, we performed a whole-brain analysis contrasting the ASD and control groups in each run of the neurofeedback session, followed by seed-based connectivity analysis, taking the subject-specific DLPFC target ROI as a seed. In this way, we tried to disentangle how differences in brain activity between groups and along the neurofeedback session (within-group) might be promoted by changes in connectivity between the target ROI (DLPFC) and other brain regions, particularly evaluating the gained ability to adequately activate the target region even when feedback is absent (transfer run).

In whole-brain analysis, we found significant differences only during the pre-neurofeedback and the first neurofeedback runs, again with an apparent “similarity” of ASD brain activity pattern to the control group on the following runs, including the final transfer run. During the pre-neurofeedback run, a single cluster from the ASD>control contrast emerged, localized in the left premotor cortex. Sensorimotor dysfunction has been recognized in ASD since the first descriptions and, clinically, even emerges before the development of core features [52]. These altered complex motor functions include deficits in movement preparation/planning and stereotyped/repetitive behaviors (repetitive hand flapping, rocking), which are dependent on frontostriatal loops. Thus, it has been stated that executive dysfunction theory may account for such complex motor features [3, 6, 53]. A recent meta-analysis of EF fMRI studies in ASD supported this suggestion, showing less recruitment of sensorimotor areas while performing EF tasks (compared to controls) [16]. Differences in motor areas, including the premotor cortex, were also demonstrated by other neuroimaging techniques, such as spectroscopy (reduced GABA concentration) [54] and tractography of the corticospinal tract [55]. These metrics were correlated with the severity of hyperresponsiveness and repetitive/restricted behaviors, respectively. Thus, our results suggest that EF training through rt-fMRI neurofeedback might also have a positive impact on these sensorimotor areas. Indeed, this hypothesis is reinforced by the functional connectivity analysis that reveals an increase in connectivity between DLPFC and motor/somatosensory cortex from pre-neurofeedback to transfer run in the ASD group, which might indicate a compensation of the functional deficit in motor areas found in the whole-brain analysis of pre-neurofeedback run and absent in transfer run, promoted by neurofeedback.

On the other hand, the comparison in the control group (transfer>pre-neurofeedback) showed decreased bilateral connectivity with the parahippocampal gyrus, possibly associated with a diminished dependence on working memory imagery in long-term memory after neurofeedback training.

When contrasting the ASD and control groups in transfer runs, we found clusters of hyperconnectivity with the DLPFC in the left SFG, left posterior MFG, left MTG, left mPFC, and right occipital pole. Higher functional connectivity in the left MFG and SFG represents short-range connectivity within dorsolateral prefrontal areas (but outside the defined target ROI), functionally linked as the executive core. On the other hand, the mPFC and MTG have important roles in executive processes, the former integrating new information and the latter being the core of conceptual processing and mediating integration of information from the DMN and multidemand/or executive network [56, 57]. The ventral mPFC (vmPFC) also has a supportive role in learning associations between emotional arousal and feedback [47]. A previous trial in overweight/obesity for volitional modulation of DLPFC activity showed higher DLPFC-vmPFC connectivity with neurofeedback training, denoting improved self-control [58]. In ASD, decreased activation in the mPFC [59] reduced functional connectivity between the mPFC and premotor and somatosensory cortex [60] and reduced effective connectivity between the mPFC and DLPFC was reported [61]. Thus, the increased connectivity between DLPFC and these areas might, again, reflect a connectivity-based compensatory mechanism induced by neurofeedback training.

The cluster of hyperconnectivity found in the occipital pole merits due consideration. A few theories support abnormal visual processing in autism, such as the weak central coherence theory [62]. This theory considers that an enhanced ventral stream and defective dorsal stream of visual processing might partially account for ASD symptomatic picture, in particular the “islets” of perceptual competence. Additionally, reduced functional connectivity between the frontal and visual cortices has been reported in ASD [63]. Thus, an increase in DLPFC-visual cortex connectivity in the transfer run may represent, again, another dysfunctional mechanism in ASD being probed by neurofeedback.

Finally, between-group whole-brain analysis considering the first neurofeedback run showed a higher PSC in the precuneus and angular gyrus, both parts of the DMN, for the ASD group. Since the precuneus and angular gyrus integrate the default mode network (DMN) and consistently show deactivation during imagery tasks in both groups (independent of feedback), we interpret this positive difference in the ASD group as lower deactivation compared to controls. The DMN and executive control networks have a well-known anticorrelation [64, 65], and deactivation of the DMN during cognitively demanding tasks has also been demonstrated in the context of neurofeedback [50]. Moreover, previous studies with resting-state fMRI reported a predictive value of posterior cingulate cortex/precuneus connectivity metrics for neurofeedback [66, 67]. Thus, we understand these differences in whole-brain activation patterns between groups in the first neurofeedback run mainly as a reflection of neurofeedback performance itself (not related to specific imagery tasks), with a lower adaptation of ASD to this type of training in the first contact but being rectified in the following runs.

A possible limitation of our study is that our control group is not demographically matched and differs from the ASD group in age and gender. Since the prefrontal cortex maturation continues into adulthood [68, 69], age can be a potential confounder in our results. However, previous metanalyses in ASD suggested that impairments in working memory/EF do not correlate with age and that fMRI activations are consistent from childhood to adulthood [3, 16]. Concerning gender, in the neurotypical population, various studies, from small samples to meta-analyses, failed to show differences in brain activation during verbal working memory tasks [36, 70, 71]. However, this might not be the case in ASD, where gender apparently influences neurofunctional patterns and phenotype [72–75], justifying the option for exclusive male samples in all the rt-fMRI neurofeedback studies published thus far. Further research intended to prove the therapeutic effect of neurofeedback in larger samples and allowed to be more heterogeneous, should include females and a wider age range (namely, children). As an alternative, future studies with only females will be very important while avoiding heterogeneity. Finally, we did not assess the intelligence quotient of the neurotypical participants, so we cannot exclude a potential influence of this aspect in the results.

An additional confound in our data is the interscanner variability, since the research project was interrupted by an MR upgrade. We tried to minimize this problem by always considering the PSC relative to the ‘Baseline’ condition in both the RFX GLM whole-brain analysis and the connectivity analysis between groups. Additionally, we supported our interpretation not only in between-group results but also in integrating the functional brain patterns along the runs within each group.

The goal of this proof-of-concept study was to investigate the ability of the DLPFC to perform self-modulation and the underlying mechanisms of the neurofeedback response in ASD. We did not focus on behavioral/clinical changes, since this would be hard to achieve in a single-session neurofeedback protocol. Additionally, considering that we did not intend, at this point, to prove therapeutic effect, we have a modest total sample size (n=29), larger than the median of neurofeedback studies (n=20) and powered to detect medium to large effect sizes [51], but limiting the interpretation of our results outside the target ROI.

Overall, we consider our results to be encouraging enough to proceed to a clinical trial of EF enhancement in ASD through rt-fMRI neurofeedback.

## Conclusions

In this study, we investigated for the first time the potential of rt-fMRI neurofeedback for executive function enhancement in ASD. First, we demonstrated the feasibility of our working memory neurofeedback paradigm, with all participants being able to modulate the DLPFC with imagery, from both ASD and neurotypical groups. However, in runs without neurofeedback, the DLPFC activity level was significantly inferior in ASD, suggesting that feedback probes the mechanisms of DLPFC activity modulation. Furthermore, when comparing the whole-brain functional pattern, we found that groups solely differed in pre-neurofeedback and first neurofeedback run, including premotor and DMN regions, becoming similar along the train. Finally, we looked into connectivity between DLPFC and other brain regions, reflecting the influence that the trained modulation of this specific area has in a wider neural network. Here, we found higher connectivity between DLPFC and motor areas in ASD in the transfer run compared to the pre-neurofeedback run. We also showed increased connectivity between DLPFC and several areas, including left MFG, left MTG, left ventral mPFC, and right occipital pole, specifically in the ASD group. We propose that this might reflect a reorganization of connectivity between DLPFC and low-level areas and other high-level areas, both reported to be implicated in autism underlying neurobiology [76, 77], promoted by neurofeedback. Our results encourage the reproduction of this neurofeedback paradigm in a larger sample,with a multisession protocol, also evaluating behavioral and clinical impact, to better establish the value of this type of training for executive functioning improvement in ASD.

### Supplementary Information

**Supplementary Material 1.**

**Supplementary Material 2.**

**Supplementary Material 3.**

## Data Availability

The datasets used and/or analyzed during the current study are available from the corresponding author upon reasonable request.

## Abbreviations

ACC: Anterior cingulate cortex
ASD: Austism spectrum disorder
DLPFC: Dorsolateral prefrontal cortex
DMN: Default mode network
EF: Executive function
IFG: Right inferior frontal gyrus
MDN: Multidemand network
MFG: Right middle frontal gyrus
MTG: Middle temporal gyrus
mPFC: Medial prefrontal cortex
PSC: Percent of signal change
ROI: Region of interest
rt-fMRI: Real time functional magnetic resonance imaging
SFG: Superior frontal gyrus
SMA: Supplementary motor area
