# Supplementary material for: Neurofeedback training of executive function in autism spectrum disorder: distinct effects on brain activity levels and compensatory connectivity changes
Source: J Neurodev Disord. 2024 Apr 11;16:14. doi: 10.1186/s11689-024-09531-2 (PMC11008042; doi:10.1186/s11689-024-09531-2)
Supplement: Supplementary file 1 — Supplementary Material 1. [file 11689_2024_9531_MOESM1_ESM.pdf]

CRED-nf checklist summary

20 May, 2023

Manuscript title: Neurofeedback training of executive function in Autism Spectrum Disorder: distinct effects on brain activity levels and compensatory connectivity changes

Corresponding Author: Daniela Jardim Pereira
Corresponding author email: danielajardimpereira@gmail.com

Item No.	Checklist item	Manuscript Details
**Pre-experiment**		
1a	Pre-register experimental protocol and planned analyses	*This experiment was not preregistered*
1b	Justify sample size	”we have a modest sample size (n=29), larger than the median of neurofeedback studies (n=20) and powered to detect medium to large effect sizes (Fede et al., 2020), but limiting the interpretation of our results outside the target ROI.”
**Control groups**		
2a	Employ control group(s) or control condition(s)	The rt-fMRI neurofeedback protocol was previously validated with a sham-controlled study in a neurotypical population (Pereira et al., 2023)
2b	When leveraging experimental designs where a double-blind is possible, use a double-blind	*NA: A double-blind was not appropriate for this experiment*
2c	Blind those who rate the outcomes	*Those who rated the outcome were not blind to group assignment*
	Blind those who analyse the data	*Those who analysed the data were not blind to group assignment*
2d	Examine to what extent participants and experimenters remain blinded	After the scanning session, participants answered a debriefing questionnaire that included subjective questions about their feelings during the acquisition (How did you feel during the neurofeedback session?), the contingency between effort and feedback change (Did you feel there was a correspondence between the used strategies and the given feedback?) and the used cognitive strategies (What was the maximum number of sequences you could picture in each block? And the maximum digit number? Which strategies worked better? And which ones did not work?)
2e	In clinical efficacy studies, employ a standard-of-care intervention group as a benchmark for improvement	*NA: This is not a clinical efficacy study*

**Control measures**		
3a	Collect data on psychosocial factors	*Psychosocial factors were not measured*
3b	Report whether participants were provided with a strategy	During the neurofeedback runs, participants were instructed to empty the thermometer during 'Baseline' conditions and increase the thermometer bars during the 'Imagery' condition. As a cognitive strategy to increase the number of bars in the thermometer, we recommended the participants to repeatedly try generating a sequence and then recite it backwards sub-vocally (Zhang et al., 2013), adjusting the content, length and difficulty of the sequences according to the feedback.
3c	Report the strategies participants used	According to the debriefing questionnaire, almost all participants perceived a correspondence between the given feedback and the imagery task. Most participants followed the suggested imagery task for activating the DLPFC (inverted recall of self-generated numeric sequences), but used different strategies to increase the thermometer. For example, some participants based their strategy on mental calculation, others relied on "visualization" of the generated sequence. In both groups, some participants reported that easier sequences (such as phone numbers, birthdays or repeated numbers) were the more effective strategies; on the opposite, other participants reported that the better way to increase the thermometer bars was increasing the task difficulty (for example aleatory numbers, bigger sequences, thinking fast). The reported maximum number of digits and sequences per block was not significantly different between groups, with ASD reporting 4 to 20 digits (M=9.375, SD=5.07) and 2 to 30 sequences (M=8.6, SD=8.9) and controls reporting 4 to 20 digits (M=8.1, SD=4.1) and 2 to 12 sequences (M=6.1, SD=3). Table 1 in the Supplementary Material (S2) summarizes the responses to the final debriefing questionnaire.
3d	Report methods used for online-data processing and artifact correction	We refer to our previous paper where we include precise methods for online-data processing: "The rt-fMRI neurofeedback protocol was previously validated with a sham-controlled study in a neurotypical population (Pereira et al., 2023), where we reported the experimental procedure in detail (please refer to Methods section of the previous paper) . "
3e	Report condition and group effects for artifacts	*Condition and group effects for artifacts were not measured, or not reported in the manuscript*
**Feedback specifications**		

4a	Report how the online-feature extraction was defined	Based on this data, we functionally defined the DLPFC online using the real-time fMRI software package Turbo-BrainVoyager 3.2 (TBV; Brain Innovation, Maastricht, The Netherlands. We generally considered ROIs appropriate for NF target, when PSC was at least 1%. Anatomical references were also taken into account by an expert neuroradiologist (DJP) to determine DLPFC, guaranteeing it was located anterior to premotor cortex and superior to the planes including the lateral ventricles. All targets were selected on the left hemisphere since participants were performing a verbal working memory task during imagination runs (Emch et al., 2019).
4b	Report and justify the reinforcement schedule	The goal of this proof-of concept study was to investigate the ability to perform self-modulation of DLPFC and the underlying mechanisms of neurofeedback response in ASD. We did not focus on behavioural/clinical changes, since this would be hard to achieve in a single session neurofeedback protocol.
4c	Report the feedback modality and content	Visual feedback was provided in the form of a thermometer that was updated every TR based on the mean ROI activation of the neurofeedback target selected during the localizer run. The thermometer was divided into 10 discrete levels with a maximum value of 2.5%, where each level represented a given range of percent BOLD signal change (0 for an empty thermometer and 0.25% for each level).
4d	Collect and report all brain activity variable(s) and/or contrasts used for feedback, as displayed to experimental participants	Visual feedback was provided in the form of a thermometer that was updated every TR based on the mean ROI activation of the neurofeedback target selected during the localizer run. The thermometer was divided into 10 discrete levels with a maximum value of 2.5%, where each level represented a given range of percent BOLD signal change (0 for an empty thermometer and 0.25% for each level).
4e	Report the hardware and software used	Data acquisition was initially conducted on a 3T Siemens Magnetom TrioTim scanner with a 12-channel head coil, which was upgraded during the period of this study for a Magnetom Prismafit scanner with a 64-channel head coil. (...) We functionally defined the DLPFC online using the real-time fMRI software package Turbo-BrainVoyager 3.2 (TBV; Brain Innovation, Maastricht, The Netherlands).
**Outcome measures - brain**		
5a	Report neurofeedback regulation success based on the feedback signal	Considering the definition of successful modulation as a significant positive t-value for the contrast of interest ('Imagery'>'Baseline'), we found that both groups were highly proficient in modulating the target region (DLPFC) using the instructed strategy. Taking into account all imagery (train, transfer and neurofeedback runs; 60 in the ASD group and 85 in the control group), we found statistically significant modulation of the target ROI in 50 runs (84%) in the ASD group and 83 runs (98%) in the control group. In the control group, the two runs without significant modulation were without feedback, meaning they achieved modulation of DLPFC in 100% of the neurofeedback runs. In ASD group, successful modulation was equally distributed in all runs (84% in each).

5b	Plot within-session and between-session regulation blocks of feedback variable(s), as well as pre-to-post resting baselines or contrasts	*The manuscript does not plot within-session and between-session regulation blocks of feedback variable(s), as well as pre-to-post resting baselines or contrasts*
5c	Statistically compare the experimental condition/group to the control condition(s)/group(s) (not only each group to baseline measures)	The group mean t-value for DLPFC in each run is represented in Figure 2, being lower in ASD group. The mixed-ANOVA considering each imagery run showed a significant effect of group in DLPFC activity (p=0.045). When separately considering differences between groups in runs with and without feedback, we found that activity in target ROI is significantly lower in ASD group (M=2.16, SD=2.21) compared to the control group (M=3.92, SD=1.82) specifically when feedback was not provided (t(27)=2.353, p=0.026).
**Outcome measures - behaviour**		
6a	Include measures of clinical or behavioural significance, defined a priori, and describe whether they were reached	*The manuscript does not include measures of clinical or behavioural significance*
6b	Run correlational analyses between regulation success and behavioural outcomes	*This manuscript does not compare regulation success and behavioural outcomes*
**Data storage**		
7a	Upload all materials, analysis scripts, code, and raw data used for analyses, as well as final values, to an open access data repository, when feasible	*No additional documents related to the materials, analysis scripts, code, raw data, or final values are available for this manuscript*
