# Supplementary material for: Neurofeedback training of executive function in autism spectrum disorder: distinct effects on brain activity levels and compensatory connectivity changes
Source: J Neurodev Disord. 2024 Apr 11;16:14. doi: 10.1186/s11689-024-09531-2 (PMC11008042; doi:10.1186/s11689-024-09531-2)
Supplement: Supplementary file 3 — Supplementary Material 3. [file 11689_2024_9531_MOESM3_ESM.docx]

**SUPPLEMENTARY MATERIAL (S2)**

| **Table 1**- Characterization of ASD and Neurotypical Groups | | | | | |
| --- | --- | --- | --- | --- | --- |
|  | **ASD** | |  | **Neurotypicals** |  |
|  | Mean (SD, range) |  | | Mean (SD, range) |  |
| Age | 22.83 (4.5; 18-32) | |  | 27.82 (4.15; 22-38) | *p=0.005** |
| Sex (M/F) | 10/0 | |  | 10/7 | *p=0.004*** |
| FSIQ | 109 (13; 92-135) | |  | - |  |
| VIQ | 112 (16; 86-144) | |  | - |  |
| PIQ | 103 (11; 80-1189 | |  | - |  |
| FSIQ – Full-Scale Intelligence Quotient; PIQ – Performance Intelligence Quotient; SD- standard deviation; VIQ – Verbal Intelligence Quotient  *independent sample t-test **Fisher’s Exact Test | | | | | |

| **Table 2. Answers to Debriefing Questionnaire** | | | | | |
| --- | --- | --- | --- | --- | --- |
| **Subject** | **1** | **2** | **3** | **4** | **5** |
| **ASD** | | | | | |
| 1 | Indifferent | Yes | 9 digits | phone numbers | aleatory choice |
| 2 | inside an armour: secure, comfortable, but with few space | Yes | maximum number of sequences 6; maximum number of digits: 5 | Both worked with their merits. When we intend to analyse brain activity, we cannot try to trivialize the general problems/solutions of an individual. | Those that were performed with a long-term intention |
| 3 | It was positive, although I felt some itchiness | yes | 20 digits per sequence | all worked, mainly imagery | all worked |
| 4 | dizziness | still trying to understand the objective of the MRI scan | don't remember | none | all |
| 5 | normal, relaxed | yes | 2 sequences, 4 digits | divisions |  |
| 6 | tired | generally, yes | 9 sequences, 6 digits | sequences previously planned (pair, odd, increasing or decreasing sequences, Fibonacci sequences) | aleatory selection of numbers |
| 7 | a little bit tired, quite afraid | yes | 5 to 8 sequences | imagine a sequence of numbers or words | sequences of two numbers |
| 8 | well | yes | 4 sequences, 9 digits each at maximum | number repetition in each sequence | using spontaneous numbers |
| 9 | well | yes | 1 to 5 | imagine easy numbers | imagine aleatory numbers |
| 10 | well, tight and cosy | yes, from what I saw feedback was positive | 30 sequences, 10 digits per sequence | think hard in sequences backwards | think sequences in snail's pace |
| 11 | frustrated because I could not get the results that I wanted | some, but little | 5 sequences, 12 numbers each | do difficult equations | think in plans |
| **HC** | | | | | |
| 1 | ok | I felt differences between imagination blocks and baseline, but quite independent on strategies | 4-6 sequences, maximum 6 digits | sequences of 5 non-consecutive numbers | none |
| 2 | Comfortable until the moment the headphones dislocated slightly, making it difficult to concentrate on the task, but it was more at end of the session | yes and it get significantly better across the experience | 5 sequences, 20 digits | known sequences which familiarity allowed to invert the direction easily | trying to increase the difficulty of the sequence, increasing the interval between digits, because when inverting the effort is bigger and attention shifts to calculation |
| 3 | unfocused on transfer run promoted by phone noises | yes in most of the used strategies for both activation and baseline runs | 9/10 sequences with 4/5 digits or 4/5 sequences with 9 digits | bigger numbers, building bigger number sequentially (5 digits and then add until 9) | numbers with less digits |
| 4 | Feedback sometimes get me distracted, there was a delay between strategy and result | yes | 10 sequences, 6 digits | visualization of numbers in screen, partially ignore feedback | concentrate too much on feedback |
| 5 | well | yes | 2 sequences, 8 digits | bigger sequences, 2 or more digits in each sequence |  |
| 6 | ok | yes, mainly with repetition if feedback runs | 3 or 4 sequences, 6 digits | generate sequences of numbers including more than 2 digits and non-monotonic, that is that not increase or decrease from beginning to end | sequences starting in 1 or with other small numbers and sequences associated to something or someone. |
| 7 | it was tiring | yes | 10-12 sequences, 7 digits | visualize numbers | sequential numbers |
| 8 | nothing in particular, some anxiety when I couldn't make the thermometer going up or down as I wanted | yes | 4/5 sequences per block with 4/5 numbers | visualize in imagination a number sequence and trace the ascending and descending pathway | imagine numbers without visual reference |
| 9 | very lonely | yes | 4 sequences, 10 digits | sequences with numbers in a row | sequences with aleatory numbers |
| 10 | normal (quite sleepy) | sometimes, but in general yes | 4 sequences, 7 digits | invert number sequence that are already know (phone numbers, birthdays). Associate each square of the thermometer to a number and invert order | invert sequences of aleatory numbers, without associate each digit to something. It went difficult for sequences with more than 4/5 digits. |
| 11 | very well | yes | 4 to 5 sequences, 5 to 8 | sequences of 15 digits imagining very quickly | think slow |
| 12 | I felt that was never really able to control feedback | I felt few correspondence between strategy and feedback. | 4 numbers, 4-6 sequences | binary sequences, sequences with repeated numbers | pre-defined sequences |

**Questions:**

1. How did you feel during neurofeedback session?
2. Did you find a correspondence between used strategies and given feedback?
3. Which was the maximum number of sequences you could picture in each block? And maximum number of digits per sequence?
4. Which strategies worked better?
5. Which strategies did not work?
